# Antimicrobial Susceptibility, Minimum Inhibitory Concentrations, and Clinical Profiles of *Stenotrophomonas maltophilia* Endophthalmitis

**DOI:** 10.3390/microorganisms9091840

**Published:** 2021-08-30

**Authors:** Ming-Chih Ho, Ching-Hsi Hsiao, Ming-Hui Sun, Yih-Shiou Hwang, Chi-Chun Lai, Wei-Chi Wu, Kuan-Jen Chen

**Affiliations:** 1 Department of Ophthalmology, Chang Gung Memorial Hospital, Linkou Branch, Taoyuan 33305, Taiwan; d4352507@yahoo.com.tw (M.-C.H.); hsiao.chinghsi@gmail.com (C.-H.H.); minghui0215@gmail.com (M.-H.S.); yihshiou.hwang@gmail.com (Y.-S.H.); chichun.lai@gmail.com (C.-C.L.); weichi666@gmail.com (W.-C.W.); 2 College of Medicine, Chang Gung University, Taoyuan 33302, Taiwan

**Keywords:** antimicrobial susceptibility, antibiotics, endophthalmitis, minimum inhibitory concentrations, *Stenotrophomonas maltophilia*

## Abstract

*Stenotrophomonas maltophilia* has been reported in various ocular infections, including keratitis, conjunctivitis, preseptal cellulitis, and endophthalmitis, all of which may lead to vision loss. However, the *S. maltophilia* strain is resistant to a wide variety of antibiotics, including penicillins, third-generation cephalosporins, aminoglycosides, and imipenem. In this study, we retrospectively reviewed the clinical characteristics, antibiotic susceptibility, antimicrobial minimum inhibitory concentrations (MICs), and visual outcomes for *S. maltophilia* endophthalmitis. The data of 9 patients with positive *S. maltophilia* cultures in a tertiary referral center from 2010 to 2019 were reviewed. Cataract surgery (*n*  =  8, 89%) was the most common etiology, followed by intravitreal injection (*n*  =  1, 11%). *S. maltophilia*’s susceptibility to levofloxacin and moxifloxacin was observed in 6 cases (67%). Seven isolates were resistant to sulfamethoxazole-trimethoprim (78%). The MIC_90_ for *S. maltophilia* was 256, 256, 256, 8, 12, 12, 12, and 8 μg/mL for amikacin, cefuroxime, ceftazidime, tigecycline, sulfamethoxazole-trimethoprim, levofloxacin, galtifloxacin, and moxifloxacin, respectively. Final visual acuity was 20/200 or better in 5 patients (56%). Fluoroquinolones and tigecycline exhibited low antibiotic MIC_90_. Therefore, the results suggest that fluoroquinolones can be used as first-line antibiotics for *S. maltophilia* endophthalmitis.

## 1. Introduction

*Stenotrophomonas maltophilia* is an aerobic, motile, opportunistic, and gram-negative bacillus that is widely distributed in soil, plants, and humid environments [1,2]. *S. maltophilia* garnered clinical attention as a nosocomial pathogen that can cause serious systemic infections, such as catheter-related bacteremia, pneumonia, and endocarditis; the pathogen is also related to prolonged hospitalization and high mortality rates, especially among patients with compromised immune systems [3,4,5,6]. *S. maltophilia* has been reported in various ocular infections, including keratitis, conjunctivitis, preseptal cellulitis, and endophthalmitis, all of which may lead to vision loss [7,8,9]. The current treatment protocol for *S. maltophilia* endophthalmitis is the intravitreal injection of antibiotics with or without pars plana vitrectomy (PPV) [10,11,12]. However, the *S. maltophilia* strain is resistant to a wide variety of antibiotics, including penicillins, third-generation cephalosporins, aminoglycosides, and imipenem [9,11]. A variable susceptibility to fluoroquinolones and vancomycin has been reported of *S. maltophilia* [1,13,14]. The multidrug resistance of the bacteria may pose a challenge for clinicians treating *S. maltophilia* endophthalmitis.

Our literature review of endophthalmitis research on PubMed revealed no specific report of minimum inhibitory concentrations (MICs) for *S. maltophilia*. Our previous study reported the antibiotic susceptibility and management outcomes for *S. maltophilia* endophthalmitis in six patients from 1998 to 2007 in Taiwan [12]. The present study focused on the same tertiary institution at which our previous study [12] was conducted and serves to update the clinical profiles, antibiotic susceptibility, and visual outcomes for a subsequent 10-year (2010–2019) period with a consecutive case series of culture-proven *S. maltophilia* endophthalmitis. Furthermore, the MIC of *S. maltophilia* was evaluated.

## 2. Materials and Methods

This single-center, retrospective study examined the data of all patients at Chang Gung Memorial Hospital (CGMH) in Taiwan with culture-proven *S. maltophilia* endophthalmitis from 1 January 2010, to 30 April 2019. The study was designed per the principles of the Declaration of Helsinki and approved by the Institutional Review Board of CGMH (IRB number: CGMH 201900614 B0 C601, 10 August 2019). Patients with a diagnosis of endophthalmitis, culture positive for *S. maltophilia,* and follow-up duration of at least 3 months were included. Demographic data, clinical presentations, interval between events and endophthalmitis diagnosis, comprehensive ocular examination results, antibiotic sensitivity, clinical management, and visual outcomes were reviewed.

All microbiological investigations were performed at the Microbiology Department of CGMH in Taoyuan, Taiwan. Bacterial culture isolates were identified using conventional microbiological methods between January 2010 and December 2013, and matrix-assisted laser desorption/ionization time-of-flight mass spectrometry performed between January 2014 and April 2019. The isolates were tested for susceptibility to multiple antibiotics by performing a Kirby–Bauer disc diffusion on a Mueller–Hinton blood agar. Clinical and Laboratory Standards Institute (CLSI; Wayne, PA, USA) standards were applied for the interpretation and quality control for each investigated year [15]. During the study period, the routine antibiotic susceptibility test for *S. maltophilia* included only levofloxacin, moxifloxacin, and sulfamethoxazole-trimethoprim (SMX-TMP).

MICs were determined using susceptibility strips (ETEST; bioMérieux, Marcy l’Etoile, France) and per manufacturer recommendations and CLSI guidelines. MIC values were determined for the following antimicrobials: tigecycline, cefuroxime, levofloxacin, SMX-TMP, gatifloxacin, moxifloxacin, amikacin, and ceftazidime.

Descriptive statistics were used to evaluate patient demographics, signs and symptoms, antibiotic susceptibility, and clinical management. Statistical analyses were performed using IBM SPSS Statistics for Windows (IBM Corp. Released 2019, Version 26.0. Armonk, NY, USA).

## 3. Results

### 3.1. Demographics of the Study Group

Table 1 presents the demographics, clinical characteristics, management, and visual outcomes of the patients in our study. The patients’ average age was 64 (range, 18–83) years. A total of nine eyes belonging to five male and four female patients were involved. The patients’ mean follow-up duration was 2.8 years (range, 3 months to 9 years). Additionally, since our hospital was a tertiary referral center, most of the cases were transferred from local clinics or hospitals. Therefore, the medical devices and contaminated solutions responsible for the possible causes of infection were hard to be identified. As far as we understood, there were no risk factors, such as contact lenses uses and keratitis, in our case series.

The causes of endophthalmitis were all exogenous and included cataract extraction (*n*  =  8, 89%) and intravitreal injection (*n*  =  1, 11%). Hypertension and diabetic mellitus were comorbid in five (56%) and four (44%) patients, respectively. Visual acuity (VA) was 20/200 in two patients (22%), counting fingers (CF) in four patients (44%), hand motions (HM) in one patient (11%), and light perception in two patients (22%). The final VA was 20/200 or better in five eyes (56%), CF in two eyes (22%), and HM in one eye (11%). One patient was lost to follow-up after a 3-month interval. Seven patients received vitreous tapping with an intravitreal injection of antibiotics (TAP) as their initial treatment, and two patients received PPV as their primary treatment. One patient (Patient 9) who underwent cataract extraction 2 months earlier received an initial diagnosis of recurrent uveitis. However, the vitreous sample from PPV indicated the growth of *S. maltophilia.* Five patients received secondary treatment, including TAP for four patients and PPV for one patient. Four patients required additional treatment.

### 3.2. Antibiotic Susceptibility and Antimicrobial Minimum Inhibitory Concentrations (MICs)

*S. maltophilia* was identified in the vitreous samples of five of the nine patients and in the anterior chamber fluid samples of four patients. The *S. maltophilia* in six isolates (67%) was revealed to be susceptible to both levofloxacin and moxifloxacin. In two isolates, *S. maltophilia* was revealed to be susceptible to SMX-TMP (22%). The clinical microbial profile of each patient is listed in Table 2.

Table 3 lists the MIC breakpoints of antibiotics. The MIC_50_ and MIC_90_ of the fluoroquinolones were as follows. The MIC_50_ and MIC_90_ of levofloxacin were 1 and 12 μg/mL, respectively. The MIC_50_ and MIC_90_ of moxifloxacin were 0.25 and 8 μg/mL, respectively. The MIC_50_ and MIC_90_ of gatifloxacin were 0.5 and 12 μg/mL, respectively.

## 4. Discussion

To the best of our knowledge, this is the first study to evaluate the MIC of *S. maltophilia* in eyes with *S. maltophilia* endophthalmitis. As in previous studies, the primary etiology of *S. maltophilia* endophthalmitis was cataract extraction. Fluoroquinolones and tigecycline exhibited a low antibiotic MIC_50_ and MIC_90_ for *S. maltophilia* isolates, whereas ceftazidime and cefuroxime exhibited high MICs. Although some *S. maltophilia* isolates exhibited resistance to fluoroquinolones, fluoroquinolones can still be an option for *S. maltophilia* endophthalmitis.

Table 4 presents the data of patients with *S. maltophilia* endophthalmitis in the literature and the present study. In the present study, the most common etiology of *S. maltophilia* endophthalmitis was cataract surgery (89%). This finding was in agreement with those of studies conducted in Argentina [2], Turkey [9], Japan [16], Germany [17], China [18,19], and the United States [20]. In our study, only one case (Patient 5) was related to intravitreal injection. Agarwal et al. [10] reported 28 cases of culture-proven *S. maltophilia* endophthalmitis caused by intravitreal injections; in their study, 23 patients exhibited a VA worse than 20/200 when they received their endophthalmitis diagnosis. However, after 17 patients received intravitreal injections with ceftazidime followed by PPV, they reported that only one patient exhibited a final VA worse than 20/200. In the present study, one patient developed endophthalmitis after receiving an intravitreal injection; however, the patient’s VA eventually improved to 20/50 after intravitreal injections of amikacin, vancomycin, and moxifloxacin and subsequent PPV and cataract extraction.

The susceptibility of *S. maltophilia* to levofloxacin has been widely reported [7,10,11,12,18,21,22]. Ji et al. [11] reported that 8 of 14 cases (57%) were susceptible to levofloxacin. In our 2010 study [12], we reported 6 cases of *S. maltophilia* ocular infection susceptible (75% to 100%) to fluoroquinolones. In the present study, we reported 6 cases (67%) susceptible to levofloxacin and moxifloxacin, indicating a greater resistance to fluoroquinolones than that found in our previous study. The higher resistance compared with the previous study may be associated with the use of intracameral and topical fluoroquinolones at the end of and after cataract surgeries in Taiwan. We also identified the MIC breakpoints of the *S. maltophilia* strain. Fluoroquinolones exhibited the lowest MIC_90_ (1, 8, and 12 μg/mL for levofloxacin, moxifloxacin, and gatifloxacin, respectively). Therefore, despite the variable susceptibilities of *S. maltophilia*, we still recommend fluoroquinolones as the first-line therapy for *S. maltophilia* endophthalmitis.

We observed high MICs for cefuroxime and ceftazidime. This finding contradicted that reported in 1997 by Hanberger et al. [23], who determined the MIC_50_ and MIC_90_ of ceftazidime to be 1 and 16 μg/mL, respectively. However, our finding may indicate that the MIC for ceftazidime has increased over this time. Because intracameral cefuroxime is widely used in prophylactic management for cataract surgery [24,25], the increased MIC may also explain the high prevalence of *S. maltophilia* endophthalmitis after cataract surgery. However, *S. maltophilia* is typically resistant to cefuroxime and has a high MIC. Although intracameral cefuroxime is often used as a prophylactic measure for preventing post-cataract endophthalmitis, cefuroxime is not an effective antibiotic for *S. maltophilia.*

Three multidrug-resistant *S. maltophilia* strains were revealed to be resistant to moxifloxacin and levofloxacin in our study, but rather low MIC_50_ and MIC_90_ of amikacin, of 4 and 8 μg/mL, were found. Given the retinal toxicity of intravitreal amikacin, amikacin is less effective for treating bacterial endophthalmitis. When a patient with *S. maltophilia* endophthalmitis exhibits no improvement after receiving intravitreal ceftazidime or fluoroquinolones, amikacin can still be used as an alternative agent.

Notably, resistance to SMX-TMP was identified in 7 of the 9 patients in our study group. This finding differed from that of our previous study [12], which reported that all the *S. maltophilia* isolates that were investigated were sensitive to SMX-TMP. An increase in resistance to SMX-TMP over the past decade was identified; this increase may be related to the spread of the *sul1* gene and class 1 integrins and insertion sequence common region (*ISCR*) elements linked to the *sul2* gene [26,27], which not only explain the increased resistance to SMX-TMP but also suggest treatment options for future *S. maltophilia* infections.

We observed a low MIC for tigecycline. A similar finding was reported by Wu et al. [28], who determined the MIC_90_ for tigecycline to be 1 μg/mL for *S. maltophilia*. Yue et al. [29] demonstrated that a combination of tigecycline and azithromycin can successfully inhibit the formation of an *S. maltophilia* biofilm. Given the results of our study, tigecycline may be another effective treatment option for *S. maltophilia* infections.

PPV is performed when a patient’s ocular inflammation persists, or the patient’s clinical condition worsens after intravitreal antibiotics. Horio et al. [16] presented two cases of endophthalmitis after intraocular lens implantation; both patients eventually received PPV and exhibited a final VA worse than 20/200. Chhablani et al. [1] also reported four patients who received PPV for dense vitreous opacities; in that study, three patients exhibited a final VA better than 20/80, and one patient’s VA remained at the light-perception level. In our study, three patients received PPV as primary or secondary treatment for severe ocular inflammation, and all patients exhibited improved VA after PPV except one excluded for loss to follow-up.

The limitations of our study are its retrospective nature and the limited number of patients who underwent antibiotic testing because of the clinical setting of the study. Because most of the cases were transferred from local clinics or hospitals, the medical devices and contaminated solutions responsible for the possible causes of infection were hard to be identified. Moreover, the inclusion of all patients with positive cultures could have introduced selection bias due to the exclusion of false-negative results. However, our study clarified the etiology, antibiotic susceptibility patterns, MICs, and visual outcomes relating to endophthalmitis caused by *S. maltophilia.*

## 5. Conclusions

*S. maltophilia* endophthalmitis, which mostly occurs after cataract surgery, is associated with a promising visual outcome when proper clinical management is implemented. Although the investigated *S. maltophilia* isolates were not fully susceptible to fluoroquinolones, fluoroquinolones were revealed to have lower antibiotic MICs than other antibiotics. Therefore, the results suggest that fluoroquinolones can be used as first-line antibiotics for *S. maltophilia* endophthalmitis.

## Figures and Tables

**Table 1:** Demographics, Clinical Characteristics, Management, and Visual Outcomes of Patients with *Stenotrophomonas maltophilia* Endophthalmitis.

No.	Age/Sex	Cause (Days)	Systemic Diseases	Initial VA	Primary Treatment (Intravitreal Agents)	Secondary Treatment (Intravitreal Agents)	Additional Treatment (Intravitreal Agents)	Final VA
1	83/M	Cataract (11)	CAD	LP	TAP (VAN + CAZ)	TAP (CAZ + AMK)		HM
2	64/M	Cataract (4)	DM, HTN, ESRD	CF	TAP (VAN + CAZ)			20/50
3	68/F	Cataract (3)	DM, HTN	CF	TAP (VAN + CAZ)	PPV (VAN + MOX + AMK)	PPV (MOX + CAZ)	Loss of FU
4	71/F	Cataract (20)	DM, HTN	CF	TAP (VAN + CAZ)			CF
5	57/M	IVI (3)	DM, HTN, MDD	HM	TAP (VAN + AMK)	TAP (MOX)	PPV + CE	20/50
6	68/M	Cataract (3)		LP	PPV (VAN + AMK)	TAP (VAN + CAZ)	PPV (VAN + AMK)	CF
7	74/F	Cataract (6)	HTN, cervical cancer	20/200	TAP (VAN + AMK)			20/40
8	76/M	Cataract (15)	DM, HTN	20/200	TAP (VAN + AMK)			20/40
9	18/M	Cataract (29)		CF	PPV *	TAP (MOX)	TAP (MOX)	20/200

Abbreviations: AMK: amikacin, CAD: coronary artery disease, CAZ: ceftazidime, CE: cataract extraction with intraocular lens implantation; CF: counting fingers, DM: diabetic mellitus, ESRD: end-stage renal disease, F: female, FU: follow-up; HM: hand motions, HTN: hypertension, IVI: intravitreal injection, LP: light perception, M: male, MDD: major depressive disorder; MOX: moxifloxacin, PPV: pars plana vitrectomy, TAP: tap and injection, VA: visual acuity, VAN: vancomycin. * The patient was initially diagnosed with having recurrent uveitis.

**Table 2:** Antibiotic susceptibility results of *Stenotrophomonas maltophilia*.

No.	Levofloxacin	Moxifloxacin	SMX-TMP
1	S	S	R
2	S	S	R
3	S	S	R
4	S	S	R
5	S	S	S
6	R	R	R
7	R	R	R
8	R	R	R
9	S	S	S

S: susceptible, SMX-TMP: sulfamethoxazole-trimethoprim, R: resistant.

**Table 3:** Minimum Inhibitory Concentration for *Stenotrophomonas maltophilia*.

MIC (μg/mL)	0.125	0.25	0.5	1	2	4	8	12	32	256
Levofloxacin			3	2 ^a^				3 ^b^		
Moxifloxacin	2	3 ^a^					3 ^b^			
Gatifloxacin		1	3 ^a^	1			1	2 ^b^		
Tigecycline			1	2	2 ^a^		3 ^b^			
Cefuroxime										8 ^ab^
SMX-TMP			1					7 ^ab^		
Amikacin					1	4 ^a^	2			1 ^b^
Ceftazidime							1			7 ^ab^

MIC: minimum inhibitory concentration, ^a^ MIC_50_ values, ^b^ MIC_90_ values, SMX-TMP: sulfamethoxazole-trimethoprim.

**Table 4:** Comparison of *Stenotrophomonas maltophilia* Endophthalmitis in the Literature and the Present Study.

No.	Author	Nationality	Year	No. of Eyes	Etiology (No. of Eyes)	Initial VA Worse Than 20/200 (%)	Fluoroquinolone Susceptibility (No.,%)	PPV	Final VA Worse Than 20/200 (%)
1	Chauhdry et al. [20]	USA	1996–1999	4	Cataract	3 (75)		2	1 (25%)
2	Chang et al. [21]	USA	1996–2005	8	Cataract	6 (75)	Levofloxacin (3, 38%)	3	2 (33%)
3	Chen et al. [12]	Taiwan	1998–2007	6	Trauma (2), Cataract (2), PK (1), Vitreous lavage (1)	6 (100)	Levofloxacin (2/2, 100%) Moxifloxacin (2/2, 100%) Ciprofloxacin (3/4, 75%)	4	2 (33%)
4	Horio et al. [16]	Japan	1999	2	Cataract	2 (100)		2	2 (100%)
5	Horster et al. [17]	Germany	1999	26	Cataract	13 * (50)		21	5 * (19%)
6	Lai et al. [3]	Hongkong	2001	1	Trauma	1 (100)		1	0
7	Karakurt et al. [9]	Turkey	2004	6	Cataract (5) Cataract+ Trab+ PPV (1)	6 (100)		2	2 (33%)
8	Chhablani et al. [1]	India	2007–2012	4	Endogenous	4 (100)	Gatifloxacin (3, 75%) Moxifloxacin (4, 100%)	4	1 (25%)
9	Ji et al. [11]	China	2010–2011	14	Cataract	10 (71)	Levofloxacin (8, 57%)	11	1 (7%)
10	Williams et al. [2]	Argentina	2014	3	Cataract	2 (67)		2	1 (33%)
11	Agarwal et al. [10]	India	2016	28	IVI	23 (82)	Levofloxacin (28, 100%)	23	1 (4%)
12	Chen et al. [18]	China	2019	4	Cataract	4 (100)	Levofloxacin (3, 75%)	4	0
13	Ho et al. (current study)	Taiwan	2011–2019	9	Cataract (8), IVI (1)	6 (75)	Levofloxacin (5, 63%) Moxifloxacin (5, 63%)	4	3 (38%)

Abbreviations: Trab: trabeculectomy, PPV: pars plana vitrectomy, PK: penetrating keratoplasty, IVI: intravitreal injection, VA: visual acuity, * VA < 20/100.

## Data Availability

The data analyzed during this study are available on request from the corresponding author, Kuan-Jen Chen. The data are not publicly available due to containing information that could compromise the privacy of the research participants.
